# Supplementary figures and images for: Academic discourse on ChatGPT in social sciences: A topic modeling and sentiment analysis of research article abstracts
Source: PLoS One. 2025 Oct 14;20(10):e0334331. doi: 10.1371/journal.pone.0334331 (PMC12520389; doi:10.1371/journal.pone.0334331)

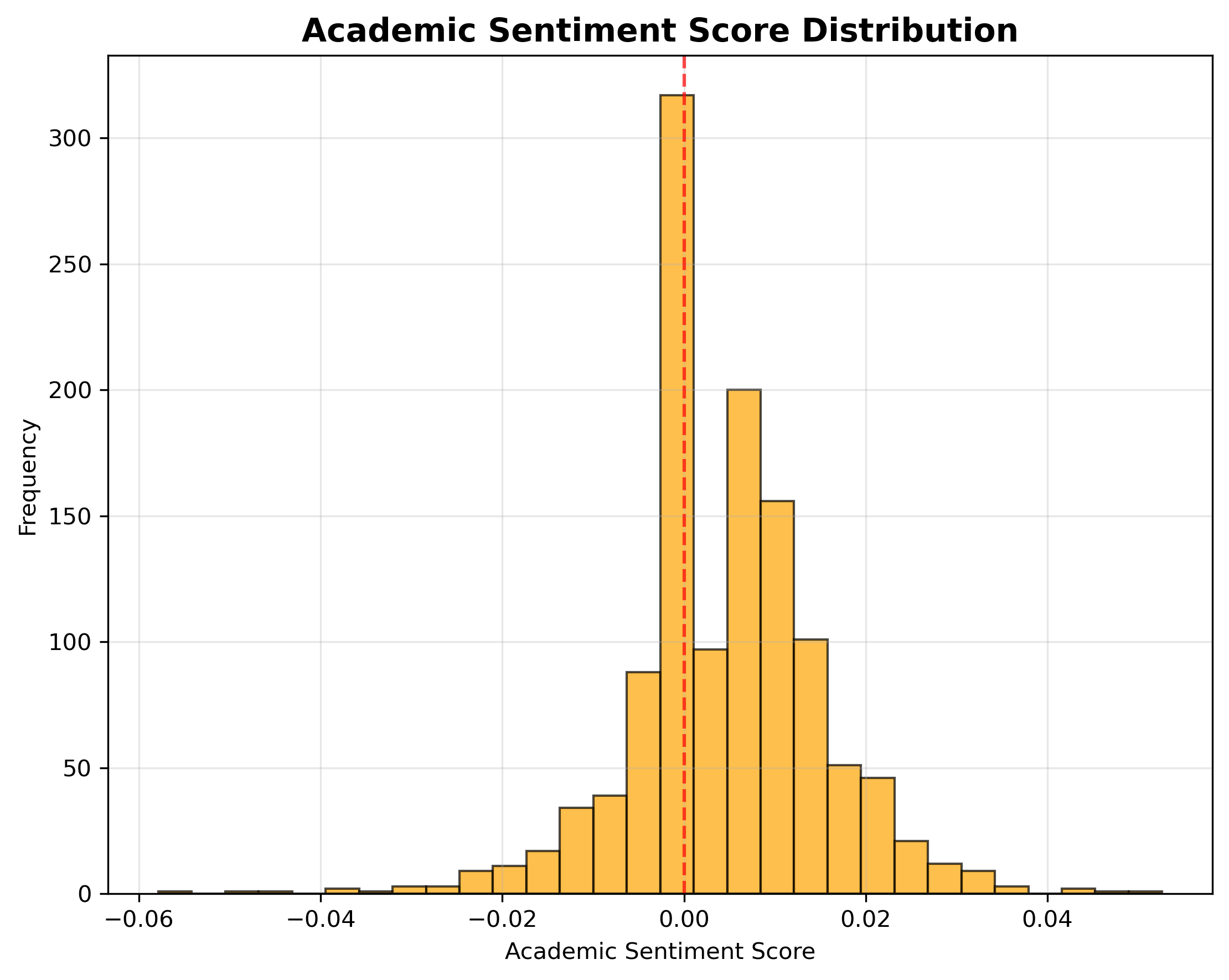

Supplement: S4 Fig — Note: All datasets and scripts have also been archived and are publicly available at Figshare: https://doi.org/10.6084/m9.figshare.29625773. (PNG) [file pone.0334331.s004.png]
