# Supplementary material for: Academic discourse on ChatGPT in social sciences: A topic modeling and sentiment analysis of research article abstracts
Source: PLoS One. 2025 Oct 14;20(10):e0334331. doi: 10.1371/journal.pone.0334331 (PMC12520389; doi:10.1371/journal.pone.0334331)
Supplement: Attachment — Submitted filename: Response to Reviewers.docx [file pone.0334331.s006.docx]

### **Dear Editors and Reviewers,**

### We thank the editor and all reviewers for their thoughtful and constructive feedback. Their comments have greatly improved the clarity, focus, and overall quality of our manuscript.

### In the following response, we address each comment point by point, organized by reviewer and numbered for clarity. Each response includes: the original reviewer comment, our detailed reply, and a description of the corresponding revisions made to the manuscript, with section and line references where appropriate. In cases where a suggestion could not be fully implemented, we provide a rationale while remaining respectful of the reviewer’s intent.

For ease of navigation, this response letter is organized by reviewer. Each section begins with the reviewer number and addresses all comments in order. For ease of reference, the starting page numbers for each reviewer’s section are listed below:

### Reviewer #1 - Page 2

Reviewer #2 - Page 8

Reviewer #3 - Page 13

Reviewer #4 - Page 25

Reviewer #5 - Page 32

### **Reviewer #1**

We sincerely thank you for the thoughtful and constructive feedback. Your comments have been extremely helpful in clarifying the focus of our study and improving the quality of the manuscript. We carefully considered each suggestion and revised the manuscript wherever appropriate. In cases where specific recommendations could not be fully implemented, we provide a rationale for our decision while remaining respectful of the reviewer’s intent. Below, we provide detailed, point-by-point responses to all comments, with corresponding revisions indicated in the manuscript.

### **Comment 1:**

### “*It would be helpful to include more details about the parameters α and β in the LDA topic modeling section. These parameters can influence the results of topic extraction, so clarifying them would help other researchers better understand and replicate your methods.*”

### **Response to Comment 1:**

### Thank you very much for this constructive suggestion. We agree that the specification of α and β is important for ensuring the transparency and reproducibility of our topic modeling procedure. In response, we have added a detailed explanation of these hyperparameters, including their specific values and rationale for selection. We now clarify that α was set to 50/K (with K as the number of topics) and β was set to 0.1, which are widely recommended defaults that support stable and interpretable results across varied corpora.

### **Manuscript Revisions**:

### A new sentence has been added on **Lines 267–271** of the revised manuscript:

### “In our implementation of the LDA model, we adopt commonly recommended hyperparameter values to ensure stable and interpretable results: α is set to 50/K, where K is the number of topics, and β is set to 0.1. These settings are in line with standard practices suggested by [61] and are known to balance topic sparsity and generalization effectively across diverse corpora.”

### **Comment 2:**

### “*While the NRC EmoLex dictionary is widely used in sentiment analysis, it wasn’t specifically designed for academic texts. It would be good to discuss its applicability and limitations in this context.*”

### **Response to Comment 2:**

### We appreciate this important observation. In response, we have expanded the discussion in Section 3.3 to acknowledge that the NRC Emotion Lexicon was originally developed for general-purpose and social media texts. We now clarify that, although the lexicon may not fully capture the subtleties of sentiment in academic discourse, it remains a suitable tool for identifying broader evaluative tendencies across documents, which aligns with the primary goal of our sentiment analysis. To further address this limitation, we have also included an explicit note in the Limitations section (Section 5.1), where we acknowledge genre-specific constraints and report that we conducted a supplementary validation using a domain-sensitive academic lexicon. This helps to strengthen the robustness of our sentiment interpretation.

### **Manuscript Revisions:**

### **Section 3.3 (**Lines 311–318**):**

### “Although the NRC lexicon was originally developed for general-purpose and social media texts, it has been adopted by [78] to score sentence-level positivity/negativity in life science abstracts. While we acknowledge that its application to academic abstracts may not fully capture the subtlety of sentiment expression in academic discourse, this limitation is less significant in our case. Our goal is not to detect affective cues at the sentence level, but rather to uncover broader evaluative tendencies across academic abstracts. From this perspective, the NRC lexicon remains a useful and interpretable tool for sentiment analysis across documents.”

### **Section 5.1 Limitations (**Lines 806–810 **):** Acknowledged potential genre-specific biases in NRC-based sentiment analysis.

### **Comment 3:**

### “*There could be more analysis on how Topic 6 (on computational processes and LLMs) connects with other topics*.”

### **Response to Comment 3:**

### We appreciate this suggestion and have addressed it in our response to **Comment 11**. In particular, we added a topic network graph and discussed how Topic 6 relates to other themes such as technological communication and education (see newly added Section 4.2.3 and Fig 7).

### **Comment 4:**

### “*While ‘trust’ as an emotion dominates across all topics, I think it would be useful to explore why this is the case. This could be tied to the objectivity required in academic writing, and explaining this would help clarify the findings.*”

### **Response to Comment 4:**

### Thank you for raising this thoughtful point. We agree that the prominence of the emotion “trust” warrants further reflection. In response, we have expanded Section 4.4.2 to provide a contextual interpretation of this pattern. Specifically, we now discuss how trust can be understood as an epistemic emotion commonly embedded in academic discourse, particularly within abstracts that emphasize methodological soundness and conceptual legitimacy. We also offer an alternative interpretation, suggesting that the dominance of trust may reflect the academic community’s confidence in ChatGPT’s potential utility and reliability. These additions aim to clarify the evaluative orientation of scholarly communication in our corpus.

### **Manuscript revisions:**

### Section 4.4.2 (Lines 716-727):

### “... In this context, trust functions as an epistemic emotion that aligns with the academic imperative to demonstrate methodological rigor and conceptual legitimacy. This aligns with prior studies showing that academic writers often adopt epistemic attitudes to establish credibility and present their claims as trustworthy [15-17]. Another possible explanation of trust as the most dominant emotion across topics might be that it indicates the academia’s confidence in ChatGPT’s reliability and utility...”

### **Comment 5:**

### “*You should acknowledge the limitation of analyzing only abstracts instead of full texts. While abstracts provide a quick overview, they might miss important details, which could affect the comprehensiveness of the results.*”

### **Response to Comment 5:**

### We appreciate the reviewer’s suggestion to acknowledge this methodological limitation. In the revised manuscript, we have added a statement in the Limitations section (Section 5.1) recognizing that academic abstracts, while commonly used in prior sentiment analysis research, possess a constrained rhetorical structure that may limit the expression of affective or attitudinal signals. We agree that this could influence the granularity and interpretive depth of our findings, and we now explicitly highlight this issue as a boundary of the present study.

### **Manuscript Revisions:**

### Section 5.1 Limitations (Lines 812–814):

### “Second, although academic discourse has been used in prior studies as valid sources for sentiment analysis, the constrained rhetorical structure of abstracts may limit the expression of affective or attitudinal cues.”

### **Comment 6:**

### “*There’s also no discussion about validating the topic modeling results. It would be beneficial to include an evaluation of topic consistency or even some manual validation to ensure the topics are representative and coherent.*”

### **Response to Comment 6:**

### Thank you for highlighting this important methodological consideration. In response, we have added a quantitative evaluation of topic model quality using the coherence score, which measures the semantic consistency among the top words within each topic. As shown in the newly added Fig 5, we plotted the coherence scores across multiple topic numbers. The six-topic solution corresponds to the highest coherence score (0.382), suggesting that it offers the most semantically coherent and interpretable topic configuration without over-fragmentation. This provides empirical support for the robustness of our topic model selection. We have incorporated a detailed explanation of this process into the revised manuscript.

### **Manuscript Revisions:**

### A newly added Fig 5 : Coherence score across different numbers of topics.

### Section 4.2.1 (Lines 384–393): A newly added paragraph to describe Fig 5.

### **Comment 7:**

### “*To broaden the research scope of the related work contents in this article, if the author thinks it is convenient, please cite the following valuable literature (which are all with the topic of sentiment analysis): [1] WordTransABSA… [2] Back to common sense… [3] Using Masked Language Modeling…*”

### **Response to Comment 7:**

### Thank you for recommending these valuable and timely references. We have now cited all three suggested works in the revised literature review (Section 2.2) to further broaden the scope of recent developments in sentiment analysis. These studies reflect advances in aspect-based sentiment analysis, particularly through the use of masked language modeling and the integration of lexical knowledge bases for affective token prediction and semantic enrichment. We appreciate the opportunity to strengthen the background section with these contributions.

### **Manuscript Revisions:**

### Section 2.2(Lines 146–149):

### “More recent studies have further enhanced aspect-based sentiment analysis by reframing it as a masked language modeling task for improved affective token prediction [37, 38] and by integrating descriptive lexical knowledge from resources like the Oxford Dictionary to enrich semantic representation [39].”

### **Comment 8:**

### “*The section on public attitudes toward chatbots (Section 2.3) could be organized more clearly. I suggest grouping the studies by platform type (e.g., Twitter, Reddit, Weibo) or sentiment (positive, negative, neutral) for a more systematic review.*”

### **Response to Comment 8:**

### Thank you for your helpful suggestion to improve the clarity of Section 2.3. While we did not adopt a strict platform-based or sentiment-based grouping, we revised the section by organizing the literature into two coherent subsections: public attitudes in social media (Section 2.3.1) and professional perspectives (Section 2.3.2). This restructuring allowed us to reflect both the user context (general public vs. professional stakeholders) and the variation in attitudinal trends. Within each subsection, we also discussed platform-specific trends and sentiment orientations to enhance interpretability. We believe this revised structure offers a more integrated and thematically coherent overview while aligning with the underlying diversity of the literature.

### **Manuscript Revisions:**

### Section 2.3 is reorganized into two subsections:

### 2.3.1 Public attitudes in social media (Lines 164–179)

### 2.3.2 Professional perspectives on ChatGPT (Lines 181–197)

### These changes improve thematic clarity by distinguishing general user sentiment from domain-specific professional concerns.

### **Comment 9:**

### “*There is no discussion on how different versions of ChatGPT (such as GPT-3.5 vs. GPT-4) might lead to differences in academic views. This could be an important point to address*.”

### **Response to Comment 9:**

### Thank you for this valuable suggestion. While we did not directly compare academic responses to specific versions of ChatGPT (e.g., GPT-3.5 vs. GPT-4), we agree that evolving versions of generative AI tools are likely to influence scholarly perceptions and evaluations over time. To address this, we have added a future research direction (Section 5.2) that emphasizes the importance of diachronic analysis. Such an approach would allow researchers to track how sentiment and thematic trends shift in response to new developments and increased integration of ChatGPT into academic workflows. We believe this longitudinal perspective can help capture version-sensitive dynamics, including emerging concerns, adaptive strategies, or broader acceptance patterns.

### **Manuscript Revisions:**

### Section 5.2 Future Research Directions (Lines 841–845):

### “Second, a diachronic analysis examining how sentiment and thematic trends evolve over time could provide valuable insight into the shifting academic discourse on generative AI. As ChatGPT continues to evolve and its applications become more integrated into academic workflows, tracking longitudinal changes may help capture emerging concerns, adaptations, or acceptance patterns.”

### **Comment 10:**

### “*Figure 8 (the relationship between topics and sentiment) could be made clearer. I recommend considering a heatmap instead of a radar chart to better show the strength of the relationship between sentiment and topics.*”

### **Response to Comment 10:**

### Thank you for this helpful suggestion regarding data visualization. In the revised manuscript, we have replaced the original radar chart with a heatmap (Fig 10) to more clearly illustrate the intensity and distribution of emotional associations across the six topics. The heatmap allows for easier comparison of sentiment frequencies and highlights distinctive emotional profiles for each topic. We have also revised the corresponding section of the Results (Section 4.4.1) to describe the key findings from the heatmap more effectively.

### **Manuscript Revisions:**

### Fig10: A heatmap showing the distribution of eight basic emotions across six topics.

### Section 4.4.1 (Lines 687–714): The section describes the heatmap and its key patterns.

### **Comment 11:**

### “*Adding a network graph to display the connections between the six topics would also be useful to visualize the potential links among them.*”

### **Response to Comment 11:**

### Thank you for this insightful suggestion. In the revised manuscript, we have added a topic-to-topic network graph (Figure 7) to visualize the semantic relationships among the six identified topics. This also allows us to address the reviewer’s earlier suggestion regarding Topic 6’s potential connections with other topics (see Comment 3). The graph is based on pairwise topic correlations and reveals meaningful patterns of inter-topic connectivity. We interpret this structure as indicative of a three-tier research architecture: a technical foundation layer (Topic 6), a user acceptance layer (Topic 3), and an application practice layer (Topics 2 and 4). This layered configuration reflects the field’s evolution from early-stage technical validation to more mature discussions on adoption, ethics, and real-world impact. The network further illustrates how ChatGPT research increasingly integrates technological, behavioral, and societal perspectives into an interconnected ecosystem.

### **Manuscript Revisions:**

### Figure 7: A topic network graph illustrating semantic connections between the six topics.

### Section 4.2.3 (Lines 525–548): Includes a detailed interpretation of inter-topic linkages.

### **Reviewer # 2**

We sincerely thank you for the thoughtful and constructive feedback. Your comments have been extremely helpful in clarifying the focus of our study and improving the quality of the manuscript. We carefully considered each suggestion and revised the manuscript wherever appropriate. In cases where specific recommendations could not be fully implemented, we provide a rationale for our decision while remaining respectful of the reviewer’s intent. Below, we provide detailed, point-by-point responses to all comments, with corresponding revisions indicated in the manuscript.

### **Comment 1:**

### “The abstract mechanically lists methods and results without explaining the novelty or importance. You should revise the abstract to emphasize what the study contributes beyond previous public/social media studies on ChatGPT.”

### **Response to Comment 1:**

### We thank the reviewer for this valuable suggestion. In the revised abstract, we have made a conscious effort to highlight the novelty and importance of our study. Specifically, we emphasize that this work provides a discourse-level analysis of peer-reviewed academic abstracts—an approach that differs from prior research focused primarily on public opinion data such as social media or surveys. We also clarify how our combination of topic modeling and sentiment analysis offers a data-driven complement to conceptual studies, helping to uncover how scholars evaluate and frame ChatGPT within formal academic discourse. We hope this revision more clearly communicates the contribution of our study.

### **Manuscript Revisions:**

### Abstract section (Lines 11-32) has been revised.

### “... By analyzing academic discourse as reflected in research article abstracts, this study contributes a discourse-level perspective on how ChatGPT is framed, endorsed, and critically examined in the social sciences. It offers a data-driven complement to existing conceptual and survey-based investigations and draws attention to both the thematic and evaluative tendencies shaping scholarly narratives around generative AI.”

### **Comment 2:**

### “*The authors mention manual filtering of abstracts but do not provide inclusion/exclusion criteria (e.g., how irrelevant abstracts were defined). Please explicitly describe the manual screening criteria and report how many abstracts were excluded at each stage.*”

### **Response to Comment 2:**

### We appreciate the reviewer’s suggestion to clarify the data screening process. In response, we have revised the manuscript to provide a detailed description of the two-step manual screening procedure. This includes the inclusion/exclusion criteria used at each stage and the number of records removed. Specifically, we now report that 66 records were excluded due to missing or placeholder abstracts, and an additional 128 entries were removed for being non-academic, non-English, or lacking meaningful content. This filtering process reduced the initial 1,421 records to a final dataset of 1,227 abstracts.

### **Manuscript Revisions:**

### Section 3.1(Lines 224–232): Full screening procedure is added.

### **Comment 3:**

### “*There is no measure of topic coherence reported to verify whether the topics make sense semantically. You should report topic coherence scores or conduct a manual validation where human coders check the interpretability of extracted topics*.”

### **Response to Comment 3:**

### Thank you for this valuable suggestion. We have added a topic coherence analysis to validate the quality of the topic model. Fig 5 shows that the coherence score peaks at six topics (score = 0.382), suggesting that this configuration achieves optimal semantic consistency. This supports the interpretability of the selected topic structure.

### **Manuscript Revisions:**

### Fig 5: Coherence score trajectory across different numbers of topics.

### Section 4.2.1 (Lines 384–393): Includes a description of topic coherence evaluation and justification for selecting the six-topic solution based on peak coherence value.

### **Comment 4:**

### “*The manuscript briefly mentions using ldatuning metrics to select the number of topics but does not show the evaluation curves or the rationale behind choosing six topics beyond metric convergence. Please provide the full tuning plots (even in supplementary materials) and justify why 6 topics were selected instead of neighboring numbers (e.g., 5 or 7).*”

### **Response to Comment 4:**

### Thank you for pointing out the need to elaborate on topic number selection. In the revised manuscript, we use Fig 4 to present the evaluation results of four ldatuning metrics (Arun2010, CaoJuan2009, Deveaud2014, and Griffiths2004) as well as the coherence score trajectory (Fig 5).

### These visualizations reveal a consistent pattern of convergence at six topics across all metrics. Specifically, Fig 4 shows that both minimization metrics—Arun2010 and CaoJuan2009—decrease sharply from 2 to 6 topics, indicating improved topic separation and reduced overlap, while providing diminishing returns beyond that point. Among the maximization metrics, Deveaud2014 reaches a clear peak at six topics (score = 1.0), then drops significantly at seven, indicating a loss in semantic clarity with further subdivision. Fig 5 further validates this result by showing that the topic coherence score reaches its highest point (0.382) at six topics. This suggests that the six-topic solution offers the most semantically interpretable structure, balancing distinctiveness and coherence. Together, these findings provide strong empirical support for selecting six topics as the optimal configuration for this study.

### **Manuscript Revisions:**

### Figure 4 shows results from four ldatuning metrics for determining optimal topics.

### Figure 5 is added to show topic coherence score trajectory across 2–20 topics.

### Section 4.2.1 (Lines 371–393): Expanded to explain the rationale for selecting the six-topic solution, integrating evidence from both evaluation metrics and coherence analysis.

### **Comment 5:**

### “*NRC is designed mainly for general or social media text and may not fit the tone of academic abstracts, leading to biased sentiment results. You should discuss this limitation.*”

### **Response to Comment 5:**

### We thank the reviewer for this important observation. In the revised manuscript, we have added a dedicated paragraph in Section 3.3 to address this limitation. We now explicitly acknowledge that the NRC Emotion Lexicon was originally developed for general-purpose and social media contexts, and may not fully capture the subtlety of sentiment expression in academic discourse. To support its applicability in our case, we cite prior research [78] where NRC has been successfully applied to analyze sentiment in life science abstracts. We further clarify that our analytic focus is not on sentence-level affective signals, but rather on identifying broader evaluative tendencies across documents. Given this objective, we argue that the NRC lexicon remains a useful and interpretable tool for our sentiment analysis. In addition, this limitation is also noted in Limitation section (Lines 807-811).

### **Manuscript Revisions:**

### **Section 3.3 (**Lines 311–318**):**

### “Although the NRC lexicon was originally developed for general-purpose and social media texts, it has been adopted by [78] to score sentence-level positivity/negativity in life science abstracts. While we acknowledge that its application to academic abstracts may not fully capture the subtlety of sentiment expression in academic discourse, this limitation is less significant in our case. Our goal is not to detect affective cues at the sentence level, but rather to uncover broader evaluative tendencies across academic abstracts. From this perspective, the NRC lexicon remains a useful and interpretable tool for sentiment analysis across documents.”

### **Section 5.1 Limitations (**Lines 806–810 **):** Acknowledged potential genre-specific biases in NRC-based sentiment analysis.

### **Comment 6:**

### “*Figures lack statistical significance testing, emotion distributions across topics are presented visually, but no statistical tests (e.g., chi-square, ANOVA) are conducted to validate whether differences are significant*.”

### **Response to Comment 6:**

### Thank you for this helpful observation. Our study adopts a natural language processing framework oriented toward exploratory, corpus-driven analysis. Within this approach, we sought to identify thematic and evaluative patterns in academic discourse, rather than to formally test hypotheses about differences between predefined groups. As such, we did not conduct statistical significance testing to compare sentiment distributions across topics. However, we fully acknowledge the value of such inferential techniques in validating whether observed differences are statistically robust. In the revised manuscript, we have incorporated this recommendation into the Future Research Directions section. We suggest that future studies may enhance analytical rigor by integrating statistical testing or bootstrapping techniques to evaluate sentiment variation across themes.

### **Manuscript Revisions:**

### Section 5.2 (Lines 851–853):

### “Fourth, future research could incorporate statistical significance testing or resampling-based inference to assess whether differences in sentiment distributions across topics are statistically robust. This would strengthen the empirical validity of observed patterns.”

### **Comment 7:**

### “*Sentiment analysis lacks robustness checks, only a single pass of NRC-based analysis was performed. Please add at least one robustness check or sensitivity analysis to validate sentiment classification outcomes.*”

### **Response to comment 7:**

### We thank the reviewer for this constructive suggestion. In the revised manuscript, we have added a robustness check to validate the sentiment analysis results. Specifically, we developed a custom-built, domain-sensitive sentiment lexicon tailored to academic discourse. This lexicon was constructed with reference to prior studies on evaluative language in scholarly texts [15–17] and was supplemented by high-frequency evaluative terms observed in our corpus. Terms were categorized into positive, negative, and neutral-but-relevant groups to reflect common evaluative tendencies in academic writing. Using this lexicon, we conducted a separate sentiment scoring procedure in Python 3.10 via Jupyter Notebook. The resulting sentiment distribution revealed a modest positive skew that is broadly consistent with the original NRC-based sentiment polarity distribution, thereby supporting the robustness of our findings. Details of this validation process are reported in the revised manuscript and included in the Supporting Material (S4).

### **Manuscript Revisions:**

### Section 4.3.1 (Lines 629–640): A new paragraph describing the construction of the custom academic sentiment lexicon and its use for robustness checking.

### Supporting Material S4: Sentiment distribution results using the academic lexicon.

### **Comment 8:**

### “*This manuscript claims that scholars have an ‘optimistic and constructive’ view of ChatGPT; however, the analysis only covers abstracts, not full articles or behavior. You should tone down the strength of conclusions*.”

### **Response to Comment 8:**

### We appreciate this important comment and fully agree that the conclusions should reflect the scope and limitations of the dataset. In the revised manuscript, we have modified the phrasing of our main interpretive claims to adopt a more cautious and qualified tone. Specifically, we now describe the sentiment landscape as “potentially varied,” and use tentative language such as “may suggest” and “tend to maintain” when referring to scholarly attitudes. Additionally, we acknowledge the presence of both optimism and concern in the discourse, noting that emotions such as trust and anticipation coexist with fear and critical reflection, especially in ethics-related contexts. These changes more accurately reflect the complexity of scholarly engagement with ChatGPT and avoid overstating the general positivity of academic views.

### **Manuscript Revisions:**

### See section 5 Conclusion (Lines 791–804).

### **Comment 9:**

### “*The following recent papers related to ChatGPT should be discussed: [1] Li et al. (2025) – PLOS ONE; [2] Hosseini et al. (2023) – PLOS ONE*.”

### **Response to Comment 9:**

### Thank you for recommending these two relevant and timely studies. We have now incorporated both into the revised manuscript as part of Section 2.3.2 (“Professional perspectives on ChatGPT”). These papers expand our discussion beyond education and research, highlighting how ChatGPT is being evaluated in healthcare contexts as well. Specifically, we cite Hosseini et al. (2023) [54] to illustrate the presence of mixed sentiments among professionals in education, healthcare, and research sectors, and Li et al. (2025) [55] to exemplify the practical use of ChatGPT in analyzing patient feedback. These additions help broaden the scope of our review and emphasize the need for sector-specific implementation strategies in different professional domains.

### **Manuscript Revisions:**

### Section 2.3.2 (Lines 189–194):

### “[54] reported mixed sentiments in an exploratory survey across education, healthcare, and research… [55] applied ChatGPT and aspect-based sentiment analysis to over 500,000 patient reviews…”

### **Reviewer #3**

We sincerely thank you for the thoughtful and constructive feedback. Your comments have been extremely helpful in clarifying the focus of our study and improving the quality of the manuscript. We carefully considered each suggestion and revised the manuscript wherever appropriate. In cases where specific recommendations could not be fully implemented, we provide a rationale for our decision while remaining respectful of the reviewer’s intent. Below, we provide detailed, point-by-point responses to all comments, with corresponding revisions indicated in the manuscript.

### **Comment 1:**

### “*The sample (the abstracts) do not necessarily reflect scholars’ perceptions since the studies may not reflect perceptions at all and an abstract is a structured text describing a research result, and not necessarily a scholarly attitude or emotion (or preferably not an emotion).*”

### **Response to Comment 1:**

### We appreciate the reviewer’s thoughtful concern regarding the appropriateness of using abstracts to infer scholars’ perceptions. We fully agree that abstracts are conventionally structured and primarily serve to summarize research content. However, from the perspective of discourse-analytical research, we respectfully argue that abstracts are not merely neutral containers of information. Rather, they function as strategic rhetorical acts that encode evaluative meaning and epistemic attitudes. We have added references to relevant literature that conceptualize abstracts as strategic and evaluative texts.

### **Manuscript Revisions:**

### To address this concern, we added the following paragraph to the Introduction section (Lines 85-91):

### “While academic abstracts are typically regarded as concise and objective summaries, research suggests that they also function as strategic rhetorical acts. From a discourse-analytic perspective, abstracts not only summarize research content, but also actively position the work by highlighting its novelty, significance, and credibility. Studies by [15-17] and [18] have shown that abstracts routinely encode authorial stance, evaluative claims, and epistemic positioning. These characteristics make them an effective genre for analyzing how emerging technologies are framed and assessed within academic discourse.”

### **Comment 2:**

### “*A sentiment analysis may therefore not be the most appropriate method to apply to abstracts.*”

### **Response to Comment 2:**

### We thank the reviewer for raising this important methodological point. We agree that academic abstracts are less likely to express overt emotion than more informal or narrative texts. However, recent research has demonstrated that sentiment analysis can still detect meaningful evaluative signals in structured academic genres such as abstracts, peer reviews, and citation contexts. In addition, our use of sentiment analysis is not intended to capture strong affective language but to uncover latent evaluative orientations that collectively reflect how ChatGPT is discursively positioned in academic discourse. This approach is supported by recent literature that has applied sentiment analysis to similar academic corpora.

### **Manuscript Revisions:**

### We have revised 2.2 section (Lines 153-161) to include the following rationale and references:

### “Additionally, topic modeling and sentiment analysis have proven effective in capturing themes and evaluative patterns in academic texts. ... Sentiment analysis has similarly been applied to detect evaluative signals in randomized trial abstracts [42], full-length research articles [43], peer reviews [44, 45], and citation contexts [46]. ...”

### In addition, we added the following sentence (Lines 812-814) to the limitations section to acknowledge the constraint raised by the reviewer:

### “Second, although academic discourse has been used in prior studies as valid sources for sentiment analysis, the constrained rhetorical structure of abstracts may limit the expression of affective or attitudinal cues.”

### **Comment 3:**

### “*The title Scholars’ Perceptions of ChatGPT in Social Sciences is somewhat misleading since the analysis was applied to the abstracts of the studies. Abstracts do strictly speaking not show scholars’ perceptions of ChatGPT, they report the outcomes of studies. I would advise to adapt the title so that it fits the study content.*”

### **Response to Comment 3:**

We thank the reviewer for this valuable suggestion. We acknowledge that academic abstracts do not provide direct access to individual scholars’ internal perceptions or beliefs, but rather represent how authors choose to frame and report their research in a public, discipline-shaped discourse. To reflect this distinction more precisely, we have revised the title to better align with the nature of our data and analysis. The revised title emphasizes discourse-level patterns in abstracts rather than attributing perceptions to individual scholars.

### **Manuscript Revisions:**

### The revised title:

### “Academic Discourse on ChatGPT in Social Sciences: A Topic Modeling and Sentiment Analysis of Research Article Abstracts”

### We believe this new title more precisely aligns with the scope, data, and methodology of the study, and avoids overinterpretation regarding scholars’ personal perceptions.

### **Comment 4:**

### “*The conclusions that “the findings offer valuable insights into scholarly attitudes towards ChatGPT within the social sciences” needs more precise wording: As said abstracts do not necessarily reflect scholarly attitudes, they rather reflect outcomes of studies which should be objectively phrased (so often neutral), and therefore the conclusions cannot be supported.*”

### **Response to Comment 4:**

We thank the reviewer for pointing out the need for more precise wording in our conclusions. We fully agree that academic abstracts do not directly reveal scholars’ internal perceptions or personal attitudes. Rather, they constitute a form of public academic discourse shaped by disciplinary conventions, audience expectations, and rhetorical objectives. As prior discourse-analytic studies have shown, academic abstracts function not only as concise and objective summaries, but also as strategic rhetorical acts that encode authorial stance and evaluative positioning [15–18]. While they may not disclose private intentions or personal emotions, abstracts frequently encode evaluative and epistemic attitudes through lexical choices, modality, and framing. From this perspective, abstracts can serve as indirect indicators of scholarly stance—not in the sense of accessing internal beliefs, but as discursively constructed evaluations that reflect how authors frame, assess, and align with emerging technologies within public academic discourse.

Accordingly, we have revised the conclusion to emphasize that our study examines how ChatGPT is discursively framed and evaluated in scholarly abstracts. Rather than inferring individual scholars’ internal attitudes, it focuses on the evaluative and epistemic patterns encoded in public academic writing.

### **Manuscript Revisions:**

### The final portion of the abstract (Lines 25-32)

### “These sentiment patterns appear to vary across thematic areas ... By analyzing academic discourse as reflected in research article abstracts, this study contributes a discourse-level perspective on how ChatGPT is framed, endorsed, and critically examined in the social sciences. It offers a data-driven complement to existing conceptual and survey-based investigations and draws attention to both the thematic and evaluative tendencies shaping scholarly narratives around generative AI.”

### **Comment 5:**

### “*The abstract of the manuscript raises questions why only social studies abstracts have been included. It could have been interesting to compare abstracts from various domains (like social sciences and medical sciences or natural sciences) and compare main themes for each domain and how these differ. […] the question rises, how representative the results are?*”

### **Response to Comment 5:**

### We appreciate the reviewer’s thoughtful suggestion regarding the potential value of comparing abstracts across multiple disciplinary domains. We agree that such a comparison between social sciences and STEM-related fields could offer valuable insights into domain-specific framings of ChatGPT. However, as this study aimed to provide a focused and in-depth analysis of discourse within the social sciences, we limited our corpus accordingly. This decision was motivated by the desire to understand how ChatGPT is positioned within socially and ethically oriented academic debates, where issues such as pedagogy, policy, and human-AI interaction are particularly salient.

### Additionally, we acknowledge the limitations this focus imposes on the generalizability of our findings. We have revised the manuscript to clearly state this in the limitations section and to position our contribution as a starting point for more comparative, cross-disciplinary investigations in future work.

### **Manuscript Revisions:**

### The rationale for not disaggregating the corpus into disciplinary subfields is provided in Section 3.1 (Lines 216–223):

“...we did not further disaggregate it by subfields. This decision aligns with our overarching research goal to provide a comprehensive, cross-disciplinary overview of the ways in which social science scholarship engages with ChatGPT. Given the exploratory nature of the study, our study is not to conduct comparative analyses across individual subfields, but rather to identify general thematic and evaluative patterns emerging from the broader academic discourse.”

### We have added the following paragraph to the limitations section (Lines 820-825):

### “Third, our analysis focused on the social sciences as a whole without disaggregating results by sub-disciplines. ... The generalizability of the findings to other research communities, especially STEM-related fields, remains limited.”

### **Comment 6:**

### “*Immediately, the question is raised why the analysis was limited to abstracts only. Abstracts, in general, have a certain function to attract the readers’ interest and therefore, negative outcomes, or more nuanced information about the precise outcomes of the studies will be lacking, amongst others due to the allowed number of words and the function and systematic structure of the abstracts. I would even argue that it is rather the case that often studies with negative outcomes will not be accepted by journals which will give the abstracts a biased representation. Thus, the usefulness of the sentiment analysis can be debated.*”

### **Response to Comment 6:**

### We sincerely thank the reviewer for this insightful critique. We fully acknowledge that academic abstracts are highly structured texts, often subject to positive reporting tendencies, and may not overtly express nuanced or critical evaluations. The rhetorical function of abstracts—to highlight significance and attract reader interest within strict word limits—does indeed constrain the expression of negative outcomes or affect-laden content. We respectfully argue that sentiment analysis remains a valid and informative tool for capturing implicit evaluative tendencies within structured academic discourse. Prior research has demonstrated the applicability of sentiment analysis to a range of academic texts, including randomized trial abstracts[42], peer review reports [44-45], and citation contexts [46]. These studies indicate that even in conventionally “neutral” genres, evaluative attitudes can be empirically detected. Furthermore, as noted in discourse-analytic literature, abstracts often encode epistemic attitudes and authorial evaluation through lexical choice and framing strategies [15-18]. Although emotional intensity may be low, affective orientation (e.g., trust, concern, enthusiasm) is often conveyed implicitly. Therefore, our use of sentiment analysis does not aim to detect strong personal emotions, but rather to identify systematic evaluative patterns at the discourse level. We agree that such analysis must be interpreted cautiously and have updated our limitations section to reflect this.

### **Manuscript Revisions:**

### To address this concern explicitly, we added lines (156-161) to the 2.2 section and lines (812-814) in the limitation section.

**Comment 7:**

“*Keywords: do the keywords reflect the main topics of the study? Key in the abstract and title are the perceptions or attitudes?*”

**Response to Comment 7:**

We thank the reviewer for this helpful observation. In our revised manuscript, we have revised both the title and the abstract to remove references to “perceptions” and to more accurately reflect the study’s focus on academic discourse and evaluative framing, as conveyed through structured abstracts. This change ensures alignment between the manuscript’s scope and the terminology used throughout. In our original submission, we admit that keywords such as “perceptions” may have indeed overstated the interpretive claims of the study. However, as PLOS ONE no longer requires authors to submit keywords, the revised manuscript does not include a keyword list.

**Manuscript Revisions:**

The keyword section has been removed in accordance with current PLOS ONE submission guidelines.

The title has been revised to:

“Academic Discourse on ChatGPT in Social Sciences: A Topic Modeling and Sentiment Analysis of Research Article Abstracts”

Corresponding adjustments have been made in the abstract and throughout the text.

**Comment 8:**

“.*.. The subfields within social sciences are not clearly discussed; the methods describing developments need citations.*

*... The aim of the study is not clearly aligned with the gap identified.*

*... A motivation could be added for focusing only on the social sciences.*

*... It can be useful to provide the main research question at the end of the introduction section*.”

**Response to comment 8:**

We thank the reviewer for this set of constructive comments, which helped us improve the clarity and focus of the Introduction section. In response, we have made the following revisions:

Clarification of disciplinary scope: We now explicitly acknowledge that our study treats the social sciences as a unified corpus and that disciplinary subfields were not disaggregated. This limitation is stated in the revised manuscript (see the Limitations section), and we reflect on how this might affect the generalizability and interpretive granularity of the results.

Citations on methodological developments: We have added supporting references for the use of topic modeling and sentiment analysis in academic discourse studies[40-46].

Explicit statement of research questions: We have added a formal statement of the three research questions at the end of the Introduction section to clearly align the study’s aims with the identified research gap.

The aim of the study is revised to align with the gap identified and the motivation has been added in Introduction section.

**Manuscript Revisions:**

Clarification on lack of subfield breakdown: 3.1 section (Lines 216-223) explains why we did not disaggreate the corpus into subfiled. This issue is also acknowledged in the Limitations section (Lines 820-825).

The aim of the study is revised to align with the gap identified (Lines 71-96)

Motivation for the focus: (Lines 71-78)

Citations on methodological background: Added in 2.2 section (Lines 153-161)

Research questions: Now explicitly listed at the end of the Introduction (Lines 97-102).

**Comment 9:**

“*However, an abstract does not reflect an attitude but abstracts represent academic outcomes preferably reported on in a neutral way. Attitudes or perceptions are not necessarily included, or rather should not be included. Moreover, abstracts are structured texts with similar information in a similar style in most of the text which is contrast to the sentiment analysis most often applied to unstructured texts to catch emotions.*”
**Response to comment 9:**

We acknowledge the reviewer’s concern, which echoes earlier Comment 6 regarding the structured nature of abstracts and the appropriateness of applying sentiment analysis to such texts. We have also explicitly addressed the rhetorical constraints of abstract writing in the revised Limitations section. We hope the explanation and revisions already provided in **Comment 6** sufficiently address this recurring concern.

**Comment 10:**

*“A concern is that CDA is described as sentiment analysis but the two methods are not mutually exchangeable. A qualitative CDA often analysis text in-depth and can include a sentiment analysis. But a sentiment analysis which only looks at the tone (positive, neutral or negative) is rather limited and I would rank it rather as a quantitative than qualitative method. The details and nuances a CDA can provide can not be provided with such a limited and automated sentiment analysis. The literature review addresses some aspects but is not consistent with regarding to the call to combine quantitative analysis with qualitative CDA, while in practice a quantitative sentiment analysis is performed. The argument why the sentiment analysis is able to reflect attitudes is not convincing..”*
**Response to comment 10:**

We thank the reviewer for this important clarification. We would like to emphasize that we did not intend to equate Critical Discourse Analysis (CDA) with sentiment analysis. Nor do we position CDA as part of our methodological framework. Our references to CDA in the literature review were solely meant to illustrate how previous studies have employed topic modeling analysis in combination with discourse-oriented approaches. These citations were used to demonstrate the broader range of possible applications of topic modeling, not to suggest that our study adopts a CDA paradigm.

In contrast, our study is explicitly designed as a data-driven, exploratory analysis that relies on fully quantitative methods, specifically, Latent Dirichlet Allocation and lexicon-based sentiment analysis. We do not claim to provide the interpretive depth that CDA offers, nor do we suggest that our sentiment analysis can capture discourse-level nuances in the same way a qualitative approach might. Rather, we use sentiment signals as indicators of evaluative orientation embedded in the textual framing of ChatGPT within academic discourse.

**Comment 11:**

“*I have a sincere question regarding to what extent an analysis of abstracts of academic papers provides insights into academia’s attitudes on ChatGPT’s role in academic settings*.”

**Response to comment 11:**

We thank the reviewer for raising this thoughtful and important question. In the revised manuscript, we have carefully adjusted our terminology to avoid potentially overreaching claims. As explained in our response to **Comment 6,** we treat abstracts as rhetorical artifacts that, despite their formal constraints, can convey evaluative positioning within scholarly debates.

We emphasize that our sentiment analysis is not intended to infer scholars’ personal emotions (see **Revised Introduction: Lines 81-84**), but rather to detect patterns of epistemic commitment, endorsement, or concern as discursively encoded in abstracts. In our study, we regard the NRC emotion categories as proxies for broader evaluative orientations, rather than as literal emotional states. Specially, emotions such as trust, anticipation, and joy are often indicative of endorsement or epistemic commitment, signaling approval, optimism, or perceived usefulness of ChatGPT in academic contexts. In contrast, emotions such as fear, sadness, anger, and disgust reflect various forms of concern, pointing to perceived risks, ethical issues, or negative consequences. Surprise is interpreted in context and may signal either endorsement (unexpected benefit) or concern (unexpected risk). Thus, these sentiment categories serve as discourse-level indicators of how ChatGPT is thematically framed and evaluated in academic discourse.

**Manuscript Revisions:**

We added the following paragraph to the Introduction section (Lines 85-91) to highlight that academic abstracts can also convey evaluative attitudes and positioning. This supports our rationale for using abstracts as the object of sentiment analysis.

### “While academic abstracts are typically regarded as concise and objective summaries, research suggests that they also function as strategic rhetorical acts. From a discourse-analytic perspective, abstracts not only summarize research content, but also actively position the work by highlighting its novelty, significance, and credibility. Studies by [15-17] and [18] have shown that abstracts routinely encode authorial stance, evaluative claims, and epistemic positioning. These characteristics make them an effective genre for analyzing how emerging technologies are framed and assessed within academic discourse.”

**Comment 12:**

“*The section ends with 3 RQs of which the first can be answered based on the abstracts, although the limitation to the social sciences limits the broadness of the outcomes. However, RQ2, regarding emotions of the scholars themselves towards ChatGPT cannot be answered by analysing the abstracts. The question raised by RQ3 is not sufficiently addressed in the literature review*.”
**Response to comment 12:**
 We thank the reviewer for this thoughtful feedback. We fully agree that our original phrasing of RQ2 may have suggested an attempt to infer scholars’ personal emotions, which is not methodologically appropriate given our data source (academic abstracts). In response, we have revised all three research questions to ensure consistency with the textual genre being analyzed and with the methodological scope of our study. In particular, RQ2 has been reformulated to refer to “sentiment orientations expressed in academic abstracts”, rather than to scholars’ individual emotions. This reframing emphasizes that our sentiment analysis focuses on discursively encoded evaluation, not affective states. Regarding RQ3, we have also rephrased it to clarify that it examines the distribution of sentiment expressions across thematic areas, and we expanded the literature review to more explicitly discuss prior research that connects sentiment with topical framing in academic discourse.

**Manuscript Revisions:**

The three research questions are now presented as follows in the Introduction:

(1) What prominent thematic patterns emerge in academic abstracts on ChatGPT in social sciences?

(2) What general sentiment orientations do scholars express towards ChatGPT in these academic abstracts?

(3) How do sentiment expressions vary across the identified thematic areas in these academic abstracts?

**Comment 13:**

“*In the figures: in figure 3 filler words are used, but why are still filler words included in the results, while other filler words like ‘the’ have been removed? In that sense, figure 3 does not give really surprising results. Why is that the case? Can it be that the function of abstracts is to report on a study, and present outcomes? what can be learned from the outcomes? I would appreciate some reflection on that*.”

**Response to comment 13:**

We thank the reviewer for raising this question, and we would like to clarify a possible misunderstanding regarding the nature and purpose of Figure 3. Figure 3 is not a global word frequency visualization, nor does it represent residual filler words left from preprocessing. Instead, it is a co-occurrence network centered on the term “student”, created to explore the semantic associations and discourse context surrounding this frequently occurring term. Its purpose is to complement the high-frequency terms shown in Figure 2 (e.g., “study”, “research”, “education”) by illustrating how the concept of “student” is embedded within broader research discussions on ChatGPT.

**Manuscript Revisions:**

We added the following clarification in **4.1 section (Lines 340-347).**

“While the frequent appearance of terms such as “*study*”, “*research*”, and “*education*” in Fig 2 may partly stem from the generic conventions of abstract writing, it also suggests a meaningful research focus within the social sciences, especially in fields like education, where scholarly engagement with ChatGPT appears to be particularly active. The appearance of “*student*” and “*learn*” among the top 10 most frequent words further implies that researchers are particularly concerned with learning processes and the impact of ChatGPT on students’ learning outcomes. This interpretation is supported by the co-occurrence network shown in Fig 3...”

**Comment 14:**
“*I would suggest to add a figure with an overview of the research procedure*.”

**Response to comment 14:**

We appreciate the reviewer’s suggestion to include a visual overview of the research procedure. We fully agree that transparency and clarity are critical, and we have taken care to describe each step of the data collection and analysis pipeline in detail in the Data and methodology section. Specifically, we outline:

- the corpus construction and selection criteria
- preprocessing procedures
- topic modeling implementation and parameter tuning
- and sentiment analysis framework

We considered including a flowchart during the revision process. However, we found that a simplified figure would largely duplicate the narrative content already provided, while a more detailed diagram might risk reducing clarity by introducing visual complexity. For this reason, we opted to retain a fully textual presentation of the procedure.

Nonetheless, we remain open to adding a schematic overview should the editor or reviewers feel it would enhance accessibility for the reader.

**Comment 15:**
“*Study, research and education most frequent words. Interpretation from the authors that educational contexts may dominate the current scholarly discourse in social sciences. (p. 15) > Isn’t that because abstracts describe a research or study by definition and look forward to what can be learned from it? What is missing is more contextual information about what kind of journals / abstracts were included, more in detail than is provided currently. Since the corpus included mainly abstracts from educational sciences / social sciences, the question rises, how representative the results are?*”

**Response to comment 15:**

We agree that the prevalence of terms such as “study,” “research,” and “education” is in part attributable to the rhetorical conventions of academic abstracts. However, we also argue that the frequent co-occurrence of terms like “student” and “learning,” along with the emergence of education-related topics in our topic modeling results, points to a genuine thematic emphasis rather than a purely structural artifact. This dual interpretation has now been clarified in Section 4.1 (Lines 340–353).

Regarding the representativeness of our corpus, we have added further detail in the Data section to clarify that our dataset consists of SSCI-indexed journals spanning a broad range of disciplines within the social sciences. That said, we acknowledge that there is a disciplinary concentration due to the nature of the dataset. This limitation is explicitly addressed in Section 5.1 (Lines 826–830).

**Manuscript Revisions:**
Section 4.1(Lines 340-353): Expanded explanation of the interpretive value of frequent terms.

Section 5.1(Lines 826-830): Acknowledge the corpus composition limitation.

**Comment 16:**

“*The selection of the type of abstracts has narrowed the scope while it would have been interesting to compare abstracts from different fields, for example, but that hasn’t been done*.”

**Response to comment 16:**

We appreciate the reviewer’s suggestion regarding the value of cross-disciplinary comparison. We agree that including abstracts from other fields, such as STEM or medical sciences, would offer a valuable contrast and potentially broaden the interpretive scope of the findings. However, as our study is intended as a focused, exploratory investigation of academic discourse within the social sciences, we deliberately limited our corpus to SSCI-indexed journals in this domain. This allowed for a more coherent and contextually grounded analysis of how socially-oriented disciplines engage with ChatGPT.

We fully acknowledge that this focus constrains the generalizability of our findings. We have explicitly discussed this limitation in Section 5.1 of the revised manuscript (Lines 820–825), and we highlight cross-disciplinary comparison as a promising direction for future research (Lines 836-840).

**Manuscript Revisions:**

1. Section 3.1 (Lines 216–223) explains why we did not disaggregate the corpus by subfield.

2. Section 5.1 (Lines 820–825): Added a paragraph noting that the corpus is limited to social science journals, and that this may reinforce discipline-specific patterns while limiting generalizability to STEM-related domains.

3. Section 5.2 (Lines 836–840): Added contents highlighting cross-disciplinary comparison as a promising direction for future research.

**Comment 17:**

“*To what extent are the results an outcome of the type of journals that were selected and what social sciences research always implies: “Our findings regarding AI and technology communication (Topic 1) and human technology interaction (Topic 5) underscore the academic community’s significant focus on the social and technical dimensions of ChatGPT” AND . > “reflects a growing recognition of the need to understand AI’s social implications beyond purely technical considerations” (that is what social sciences research implies?)*.”

**Response to comment 17:**

We appreciate the reviewer’s thoughtful observation regarding the relationship between our findings and the broader orientation of social science research. We agree that themes such as education, ethics, and human-technology interaction are central to the epistemological traditions of the social sciences, and that these priorities likely influence the topical focus of the abstracts we analyzed. Nevertheless, our intention was not to present these themes as surprising or entirely novel, but to empirically substantiate their prominence through large-scale, data-driven analysis. While it may be expected that social science abstracts emphasize socially grounded perspectives on ChatGPT, we argue that our contribution lies in demonstrating how these concerns are currently articulated and evaluated across a large body of literature. We believe this offers a useful empirical baseline that can support future comparative or longitudinal research.

**Comment 18:**

“*One of the findings from the sentiment analysis is: “On the other hand, emotions such as fear, surprise, sadness, anger, and disgust are considerably less frequent” A plausible reason could be that these kind of emotions do normally not appear in abstracts which are academic texts which also can explain why trust has the highest frequency: that is something that you want to achieve with an abstract*.”

**Response to comment 18:**

We appreciate the reviewer’s insightful comment and fully agree that the rhetorical norms of academic abstract writing typically constrain the use of strongly affective or emotionally charged language. Indeed, our interpretation does not treat the low frequency of emotions such as fear, anger, or disgust as unexpected or genre-defying. Rather, our sentiment analysis aims to show that even within a formally neutral genre, evaluative language persists in patterned ways. For instance, the predominance of trust and anticipation across certain thematic areas (e.g., educational adoption) and the relative increase of fear or concern-related terms in ethics-related topics suggest that academic discourse, such as research article abstracts, still encodes differential evaluative attitudes toward ChatGPT.

**Manuscript Revisions:**

We have explicitly noted the constraints imposed by the abstract genre in the 5.1 Limitation section (Lines 812-814).

**Comment 19:**

“*Conclusion:
- As already pointed out: a scholarly discourse is something very different from scholars’ attitudes.
- Limitation of social science abstracts is addressed (good). Make the limitations a separate heading. And discuss also other limitations such as the used method and the limitation to quantitative methods while a plea is held for combining quantitative and qualitative methods.
- Suggestions for further research: why is this research not conducted? A comparison of fields would be more interesting*.”

**Response to comment 19:**

We appreciate the reviewer’s important distinction between scholarly discourse and scholars’ attitudes. However, as prior discourse-analytic studies have shown, academic abstracts serve not only as concise and objective summaries but also as strategic rhetorical acts that encode authorial stance and evaluative positioning [15–18]. While academic discourse may not directly reveal private intentions or personal emotions, it often encodes evaluative and epistemic attitudes through lexical choices, modality, and framing. From this perspective, abstracts can serve as indirect indicators of scholarly attitudes—not in the sense of uncovering internal states, but as discursively constructed evaluations that reflect how authors frame, assess, and align with emerging technologies or issues in public academic discourse.

We have also created a separate section titled “5.1 Limitations” to clearly delineate the limitations of the study. This section now explicitly discusses the rhetorical constraints of abstracts, the disciplinary concentration of the corpus, and the methodological limitations of relying solely on quantitative, lexicon-based sentiment analysis, while acknowledging the potential value of future mixed-method approaches. In response to the suggestion for further research, we have added a distinct “5.2 Future research directions” section, where we explicitly propose comparing academic discourse across disciplines as a promising next step for extending the current study. This direction builds on the reviewer’s insight and addresses the generalizability concern noted earlier.

**Manuscript Revisions:**

Introduction section (Lines 85-91): to highlight that academic abstracts can convey authors’ evaluative attitudes and positioning.

Section 5.1 Limitations (Lines 805–834): to clarify the limitation.

Section5.2 Future Research Directions (Lines 835–856): to propose comparative analysis of disciplinary discourse.

**Reviewer #4**

We sincerely thank you for the thoughtful and constructive feedback. Your comments have been extremely helpful in clarifying the focus of our study and improving the quality of the manuscript. We carefully considered each suggestion and revised the manuscript wherever appropriate. In cases where specific recommendations could not be fully implemented, we provide a rationale for our decision while remaining respectful of the reviewer’s intent. Below, we provide detailed, point-by-point responses to all comments, with corresponding revisions indicated in the manuscript.

**Comment 1:**

“*Based on the current manuscript, it appears that the study is not clearly framed either as an empirical study or as a systematic literature review*.”

**Response to Comment 1:**

We appreciate the reviewer’s observation regarding the positioning of our study. We would like to clarify that our work is not intended to be a systematic literature review. Instead, it is a data-driven empirical analysis that aims to map how the academic community thematically frames ChatGPT and articulates its evaluative attitudes toward this technology. The study draws on a large-scale corpus of SSCI-indexed abstracts and employs topic modeling and sentiment analysis to uncover patterns in academic discourse. To avoid confusion, we have revised the framing of our study in both the Introduction and Methodology sections to emphasize its empirical and exploratory nature.

**Manuscript Revisions:**

We have revised the Introduction (Lines 71–78) to explicitly position our study as a data-driven investigation rather than a systematic literature review. Specifically, we added the sentence:

“... need for data-driven analysis to map how the academic community thematically frames ChatGPT and articulate its attitudes towards this technology. To address this gap, this study adopts an exploratory research design to analyze 1,227 SSCI-indexed abstracts from the social sciences...”

**Comment 2:**

“In the literature review section, the authors primarily summarise methodological approaches (e.g., topic modelling and sentiment analysis) without adequately reviewing prior research on GAI within the social sciences.”

**Response to Comment 2:**

We thank the reviewer for highlighting this important point. In response, we have revised the literature review section to better engage with existing research on generative AI (GAI), particularly studies that investigate how ChatGPT is perceived within the social sciences. We now include a discussion of recent qualitative studies [56–59] that explore academic perspectives on ChatGPT. This addition helps to contextualize our study and highlight the empirical gap that our research seeks to address—namely, the lack of large-scale, data-driven analysis of how ChatGPT is thematically framed and evaluated in scholarly discourse.

**Manuscript Revisions:**

We have revised the Literature Review section (Lines 198–205) to include the following contents:

“... While several studies have investigated academic perspectives on ChatGPT in social science through qualitative approaches [56–59], few have empirically examined how this technology is thematically framed and evaluated in academic discourse...”

This addition strengthens the contextual background and clarifies the empirical contribution of our study.

Comment 3：

“There is considerable overlap in aims, data, and methods between this study and existing systematic reviews using NLP methods. The research questions are perceived as overly narrow.”

Response to Comment 3：

We appreciate the reviewer’s concerns regarding the positioning and scope of our research questions. In response, we have made significant revisions to the Introduction. First, we explicitly present our research questions at the outset to clarify the focus of the study. Second, we have repositioned the study as an empirical, data-driven analysis to distinguish it from prior work. Third, we revised the framing of the research questions to emphasize their relevance to both thematic structuring and evaluative attitudes in academic discourse on ChatGPT. These revisions aim to clarify our contribution and highlight the exploratory and analytical depth of our study. We have also reduced overlap in contents across the different sections to enhance coherence and improve the logical flow of the manuscript.

Manuscript Revisions:

We revised the Introduction (Lines 71–102) to:

Clarify the empirical positioning of the study.

Present the research questions more explicitly.

This restructuring enhances the overall logical flow and makes the contribution of the study more distinct.

**Comment 4:**

“*Findings are presented descriptively without deeper interpretation or integration with existing theoretical literatur*e.”

**Response to Comment 4：**

We appreciate the reviewer’s suggestion to strengthen the interpretative depth of our findings. We would like to clarify that this study is designed as a data-driven exploratory analysis, rather than one grounded in a predefined theoretical framework. As such, our aim is to inductively uncover how the academic community thematically frames and evaluates ChatGPT, and to compare these emergent patterns with existing scholarly discussions. In line with this exploratory nature, we have revised the Results and Discussion sections to include more structured commentary and comparative references. For instance, Section 4.2.4 discusses topic findings in relation to prior studies, highlighting how our results corroborate, refine, or extend existing knowledge. Section 4.3.2 provides interpretive commentary on sentiment distribution patterns. Similarly, Section 4.4.2 situates sentiment trends across different topics. We believe this comparative approach offers meaningful contributions to the literature, even in the absence of a fixed theoretical lens.

**Manuscript Revisions:**

1. A comparative literature discussion in Section 4.2.4 (Lines 549–587) to contextualize each topic’s thematic relevance.
2. Expanded interpretative analysis in Section 4.3.2 (Lines 641–679) to explore sentiment distribution trends.
3. A cross-sectional discussion in Section 4.4.2 (Lines 715–782) to analyze how sentiment interacts with topic themes and disciplinary perspectives.
4. Added subheadings throughout the Results and Discussion sections to guide readers through descriptive findings and interpretive insights.

**Comment 5:**

“*...I strongly recommend that the authors reframe this study as a systematic literature review rather than an empirical research article*.”

**Response to Comment 5:**

We appreciate the reviewer’s thoughtful recommendation to reframe the study as a systematic literature review. However, we respectfully maintain that our study is best understood as a data-driven empirical analysis, rather than a traditional systematic review. While we do analyze a large body of academic abstracts, our goal is not to synthesize existing findings or follow a structured review protocol. Instead, we apply large-scale topic modeling and sentiment analysis to uncover latent thematic patterns and sentiment orientations within academic discourse on ChatGPT.

This approach allows us to identify emergent trends and evaluative attitudes that may not be readily captured through conventional literature synthesis. In this sense, our study complements, rather than duplicates, existing reviews by offering inductive insights from the discourse itself. To clarify this distinction, we have revised the Introduction section to emphasize our empirical and exploratory orientation.

**Manuscript Revisions:**

In the Introduction (Lines 71–78), we added the following clarification:

“.. need for data-driven analysis to map how the academic community thematically frames ChatGPT and articulate its attitudes towards this technology. To address this gap, this study adopts an exploratory research design to analyze 1,227 SSCI-indexed abstracts from the social sciences...”

**Comment 6:**

“*The manuscript lacks analytical depth and integration of key or seminal studies for each identified topic.*

*Unlike other systematic reviews, this paper does not:*

*• Discuss influential works within each topic.*

*• Identify research gaps or future directions.*

*• Provide qualitative interpretation of key findings.”*

*Incorporate discussion of exemplary works, influential keywords, and ongoing debates within each topic area. Provide more critical insights into the current state of research.”*

**Response to Comment 6:**

We appreciate the reviewer’s detailed suggestions regarding analytical depth and synthesis. We would like to clarify that this study is designed as a data-driven exploratory investigation, with the aim of uncovering emergent thematic structures and evaluative sentiment trends across a large body of academic abstracts. Our focus is on mapping patterns across the discourse rather than conducting in-depth analysis of individual topics in the style of a systematic review.

Nevertheless, we have taken steps to enrich the interpretative dimension of the study. For each identified topic, we have provided representative textual excerpts from the corpus to illustrate topic features, comparative commentary that relates our findings to relevant studies, and discussion of inter-topic similarities and divergences with relevant literature (see **Manuscript Revisions in Comment 4**). These adjustments represent an effort to partially address the reviewer’s suggestions regarding critical reflection and qualitative enrichment, while remaining aligned with the exploratory nature of our methodology.

While we acknowledge that more extensive literature integration and forward-looking synthesis are hallmarks of systematic reviews, such tasks fall beyond the scope of the current study. Instead, we believe that our approach contributes value by offering a broad, empirically grounded overview of how ChatGPT is framed and evaluated in social science research.

**Comment 7：**

Compare emotional tones and thematic emphases across disciplines (e.g., education vs. sociology) in the sentiment analysis.

**Response to Comment 7：**

We thank the reviewer for this valuable suggestion. We fully agree that examining disciplinary variation—such as comparing sentiment and thematic emphases across fields like education, sociology, or communication—could yield important insights. However, our current dataset does not include discipline-specific metadata, as we focused on capturing overall trends within the broader domain of social science research. Given this limitation, we did not conduct a discipline-level comparative analysis. We acknowledge the importance of this direction and have explicitly noted it as a limitation and future research opportunity in the revised manuscript.

**Manuscript Revisions:**

1. Section 3.1 (Lines 216–223) explains why we did not disaggregate the corpus by subfield.

2. Limitations section (Lines 820-821):

“Third, our analysis focused on the social sciences as a whole without disaggregating results by sub-disciplines. As a result, potential disciplinary differences could not be explored.”

3. Future research directions (Lines 836-840):

“Building on these limitations, several promising directions for future research can be identified. First, expanding the corpus to include abstracts from STEM and interdisciplinary journals would allow for cross-disciplinary comparisons. Such an approach could help uncover whether different academic communities conceptualize and evaluate ChatGPT in divergent ways.”

**Comment 8:**

“*Revise the introduction to foreground research aims and significance more clearly; move detailed background to later sections;clearly situate the research question within the broader social science research landscape*.”

**Response to Comment 8:**

We appreciate the reviewer’s comments regarding the structure and framing of the Introduction. In response, we have significantly revised the Introduction to better foreground the significance, aim, and methodological rationale of the study. We now introduce the research problem early on, highlighting the predominance of conceptual and qualitative studies in the current literature and the need for data-driven exploration. The revised section clearly situates our study within the broader context of social science research on ChatGPT and generative AI. We have also restructured the flow of the Introduction to ensure that the research questions appear at the end of the section, following a clear rationale for the study’s design and methodological choices. These changes improve the clarity, relevance, and logical progression of the Introduction.

**Manuscript Revisions:**

In the **Introduction (Lines 71–102)**, we revised the structure to:

Clearly state the gap in the existing literature and the motivation for a data-driven approach.

Introduce the NLP methods and explain their analytical relevance.

Theoretically justify the use of abstracts as data, drawing on discourse-analytic literature.

Present the research questions at the end to clarify the study’s focus.

**Comment 9:**

“*Provide a clearer justification for selecting the six-topic LDA solution; support it by comparing coherence, testing topic stability, or evaluating semantic exclusivity*.”

**Response to Comment 9：**

We thank the reviewer for raising this important point. In response, we have added a more detailed justification for selecting the six-topic LDA solution. Specifically, we introduced a coherence score analysis to evaluate the semantic consistency of topics across different model configurations. Coherence scores assess the degree of semantic relatedness among the top words in each topic, with higher values indicating stronger internal consistency and interpretability. The coherence score in Figure 5 peaks at 0.382 when the number of topics is set to six. This suggests that the six-topic solution achieves the optimal balance between thematic granularity and semantic coherence. Increasing the number of topics beyond this point would likely lead to fragmentation of meaningful patterns into less coherent sub-themes.

**Manuscript Revisions:**

In the **4.2.1 section (Lines 384–393)**, we added a new paragraph explaining the use of coherence scores to determine the optimal number of topics. We added Figure 5 to visualize the coherence trajectory, demonstrating that the six-topic solution provides the highest score (0.382).

**Comment 10:**

“*The lexicon-based NRC sentiment analysis method has known limitations, particularly when applied to academic texts, which often employ a neutral tone even when expressing strong critique or praise. The authors should justify their methodological choice, acknowledge its limitations, and consider incorporating a more nuanced interpretation in the Discussion section*.”

**Response to Comment 10:**

We thank the reviewer for this thoughtful and constructive comment. In response, we have addressed the issue on two levels. First, in Section 3.3, we clarified the rationale for using the NRC lexicon in academic contexts. While originally developed for general-purpose and social media texts, the NRC lexicon has been adopted in prior studies to analyze sentiment in scientific abstracts (e.g., [78]). We further explained that our goal is not to detect sentiment at the sentence level, but to capture broader evaluative tendencies at the document level, which the NRC lexicon is well suited to support.

Second, we have acknowledged the limitations of the NRC lexicon in the Limitations section, noting that genre-specific language use in academic texts may affect its accuracy. To partially address this, we implemented a supplementary validation using a domain-sensitive academic sentiment lexicon.

In addition, we provided a detailed interpretation of the sentiment analysis result in Section 4.4.2, where we interpret emotional trends within each topic area in relation to academic discourse conventions, genre-specific rhetorical practices, and relevant empirical findings. For instance, we explain how the predominance of trust can be understood as an epistemic emotion aligned with scholarly norms of credibility and rigor. We also discuss how emotions such as anticipation, surprise, and fear reflect varying disciplinary orientations and topic-specific concerns, supported by recent literature. Moreover, we highlight the limited presence of negative emotions like disgust and sadness, and explain this in terms of the problem-solving tone typical of academic discourse. These interpretations aim to go beyond numerical sentiment distributions to provide a richer understanding of how evaluative attitude is encoded in scholarly writing.

**Manuscript Revisions:**

1. Added justification for NRC lexicon use in Section 3.3 (Lines 311–318), with reference to prior research on academic texts.
2. Added a robustness check of the sentiment results by using a custom-built, domain-sensitive sentiment lexicon tailored to scholarly discourse in Section 4.3.1 (Lines 629-640).
3. Included discussion of NRC’s genre-related limitations in the Limitations section (Lines 807–811).
4. Expanded Discussion in Sections 4.4.2 (Lines 715–782) to provide more nuanced interpretation of emotion distribution across topics.

**Reviewer #5**

We sincerely thank you for the thoughtful and constructive feedback. Your comments have been extremely helpful in clarifying the focus of our study and improving the quality of the manuscript. We carefully considered each suggestion and have revised the manuscript accordingly. Below, we provide detailed, point-by-point responses to all comments, with corresponding revisions indicated in the manuscript.

**Comment:**

“*I recommend publication pending one major issue that needs addressing: data limitations. The authors analyze 1,227 research article abstracts and use these as a basis for claims on how scholars assess AI. However, this data represents a nonrandom subset of scholars, those who chose to spend considerable time and energy publishing on the topic. It makes sense that such a selection effect would go along with more positive results. On page 23 (pdf) the authors note that different data sources such as twitter posts yield different sentiments towards LLMs. This is glossing over what is a major limitation/issue that should be addressed throughout the text. Claims need to be qualified with the caveat that the authors’ data is a biased sample. This issue doesn’t defeat the project or mean it’s not without value (and cool), it merely should be noted by the authors as a limitation both up front and in the discussion. For example on page 12 (pdf) the authors state one RQ as “What are scholars’ general emotions towards ChatGPT?” But aren’t examining scholars in general or a representative sample of scholars, rather we have a nonrandom sample of scholars’ sentiments. Simple fix with a qualifier added to the RQ and the rest of the paper to match what is actual done on the empirical side: What are the general emotions of scholars who publish papers on LLMs towards ChatGPT? or something similar*.”

**Response to Comment:**

We thank the reviewer for this important observation. We fully agree that the dataset reflects a self-selecting group of scholars who have chosen to publish on ChatGPT, and that this may introduce a degree of selection bias. In response, we have taken the following steps to address this concern:

We revised the title and all three research questions to specify that our analysis is based on academic abstracts rather than scholars in general. We also added a statement in the Limitations section explicitly acknowledging the non-random nature of the dataset and its potential influence on the sentiment outcomes. This includes a caution that our findings should be interpreted within the context of this self-selecting population.

We believe these revisions help ensure alignment between our empirical scope and the claims we make, and we thank the reviewer for prompting this important clarification.

**Manuscript Revisions：**

Title revised to: “Academic Discourse on ChatGPT in Social Sciences: A Topic Modeling and Sentiment Analysis of Research Article Abstracts”

Research questions revised to:
(1) What prominent thematic patterns emerge in academic abstracts on ChatGPT in social sciences?

(2) What general sentiment orientations do scholars express towards ChatGPT in these academic abstracts?

(3) How do sentiment expressions vary across the identified thematic areas in these academic abstracts?

**Limitations section (Lines 814-819)** expanded to include the following clarification:

“The dataset analyzed in this study consists of a non-random sample drawn from abstracts of researchers who have chosen to publish on ChatGPT in SSCI-indexed journals. This introduces a potential selection bias, as these researchers may hold more engaged or optimistic views compared to the broader academic community. As such, the findings should be interpreted within the context of this self-selecting group, rather than generalized to all scholars or disciplines.”
